# Asymmetric Degenerative Changes Between Convex and Concave Sides in Symptomatic Adult Degenerative Scoliosis

**DOI:** 10.3390/jcm14196825

**Published:** 2025-09-26

**Authors:** Jun-Ho Lee, Sung Hyun Lee, Eun Ah Cho, Jin Hee Ahn, Jae-Geum Shim, Eun Jung Oh, Seunghyeon Lee, Ji Na Kim

**Affiliations:** 1 Department of Anesthesiology and Pain Medicine, Kangbuk Samsung Hospital, Sungkyunkwan University School of Medicine, Seoul 03181, Republic of Korea; 2 Department of Radiology, Kangbuk Samsung Hospital, Sungkyunkwan University School of Medicine, Seoul 03181, Republic of Korea

**Keywords:** adult degenerative scoliosis, paraspinal muscle, muscle degeneration, disc degeneration, foraminal stenosis, asymmetric degenerative changes

## Abstract

**Background/Objectives**: Adult degenerative scoliosis (ADS) is a three-dimensional spinal deformity caused by asymmetric degeneration of intervertebral discs, facet joints, and paraspinal muscles (PSM). Although previous studies have identified bilateral asymmetry in the multifidus (MF), the erector spinae (ES), and psoas (PS), the underlying mechanism remains unclear. This study aims to compare muscle volume (MV), fat infiltration (FI), and degenerative changes on the concave (CC) and convex (CV) sides in symptomatic ADS patients. **Methods**: This study included patients with ADS who visited Kangbuk Samsung Hospital between 2022 and 2024. MV and FI were measured by CT scans, and facet joint degeneration (FJD), disc degeneration (DD), and foraminal stenosis (FS) were assessed by MRI. The values of CV and CC side were compared. **Results**: 74 patients were enrolled with a mean age of 71.38 ± 8.6 years. MV of all PSM was significantly lower on the CC side than CV side. FI of ES and MF was significantly higher on the CC side compared to the CV side. Degenerative changes in the intervertebral discs and neural foramen were more severe on the CC side. **Conclusions**: In patients with ADS, we found that MV was consistently lower on the CC side across all PSM, whereas FI in the MF and ES was higher on the CC side. Degenerative changes in the spine, such as FS and DD, were also more severe on the CC side, although FJD was not. These results suggest that spinal muscle and structural degeneration are not evenly distributed between two sides. Physiotherapy that specifically targets the more affected CC side may offer better outcomes for patients with ADS.

## 1. Introduction

Adult degenerative scoliosis (ADS) is a three-dimensional deformity of the spine with a coronal deviation greater than 10°. The pathophysiology of ADS results from asymmetric degeneration of the intervertebral discs, facet joints, and paraspinal muscles, primarily in the lumbar region, leading to unequal loading of the spinal column [1].

As the global population ages, the prevalence of ADS is increasing, becoming a significant public health concern. Studies have reported its prevalence to be as high as 35.9% in the population over the age of 60 [2]. With an aging population and an increased attention to quality of life versus cost issues in the current healthcare environment, degenerative scoliosis has become a considerable healthcare concern, not only cosmetically, but also as a cause of significant pain and disability [3].

The development and progression of ADS is related to muscle degeneration in paraspinal muscles (PSM), including multifidus (MF), erector spinae (ES), and psoas muscle (PS), which is one of the largest muscles attached to the vertebral column connecting the pelvis and spine. There are no definitive conclusions as to whether bilateral muscle asymmetry is the cause of ADS or, conversely, ADS causes bilateral muscle asymmetry. Several previous studies have shown consistently that bilateral asymmetry occurs in the MF. The results show that the concave (CC) part has a smaller muscle volume (MV) in the MF than the convex (CV), accompanied by degenerative changes and fat infiltration (FI) [4,5,6,7]. However, there have been few studies on ES and PS, and no consistent conclusions have been reached. The significant mechanisms of ADS pathophysiology are assumed to include facet joint degeneration, disc degenerative changes, and foraminal stenosis that occur asymmetrically, but there are no previous studies showing differences in the asymmetry of the two sides, CC and CV.

The aim of this study is to investigate spinal parameters, including muscle, facet joint, lumbar disc, and lumbar foramen, which can cause bilateral asymmetric changes, and to find out their association with symptoms.

## 2. Materials and Methods

Patient selection: ADS patients who visited Kangbuk Samsung Hospital, Sungkyunkwan University School of Medicine, between 2022 and 2024 were included in this study. The inclusion criteria were patients aged between 50 and 90 years with a Cobb angle of 10 degrees or more, measured on anteroposterior (AP) radiography of the L-spine, with L-spine CT and MR imaging. Cobb angle was measured using AP radiography of the L-spine in a standing position. The angle was determined by drawing lines parallel to the superior endplate of the upper vertebra and the inferior endplate of the lower vertebra of the scoliotic curve, with the intersecting angle measured as the Cobb angle. Patients were excluded if they had a history of scoliosis in childhood or adolescence, had undergone spinal surgery, suffered severe spinal trauma, had spinal tumors, or had any conditions that could affect muscle or spinal health. This study protocol was reviewed and approved by the Institutional Review Board of Kangbuk Samsung Hospital (IRB No. KBSMC 2025-03-048) and adhered to the Declaration of Helsinki.

**Figure 1:**
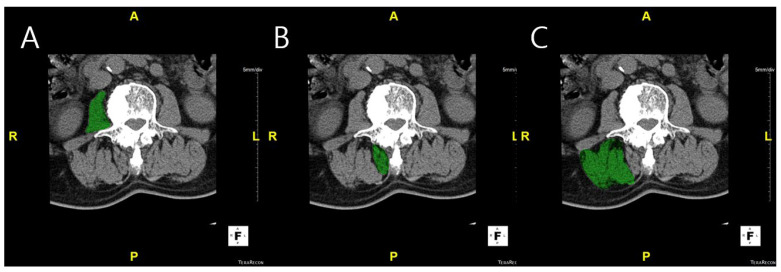
Axial CT images showing ROI of paraspinal muscles using Aquarius iNtuition Viewer (TeraRecon Inc., Foster City, CA, USA). The muscles highlighted in green indicate the segmented ROI. (**A**) Psoas muscle. (**B**) Multifidus muscle. (**C**) Erector spinae muscle.

Measurement of muscle volume and fat infiltration ratio: MV was measured using CT scans. L-spine images were obtained from the upper endplate of T12 to the lower endplate of S1, using a 256-slice CT scanner (Somatom Drive, Siemens Healthineers, Erlangen, Germany). CT examinations were performed with 2 mm collimation and 120 kV. The DICOM images were imported into Aquarius iNtuition Viewer (TeraRecon Inc., Foster City, CA, USA). Regions of interest (ROIs) were manually delineated on each axial CT slice to measure the muscle volume and fat infiltration. ROIs were drawn to include the entire cross-sectional area of the psoas, multifidus, and erector spinae muscles, carefully following the visible muscle boundary while excluding adjacent fat, bone, and other non-muscular tissues. Muscle volume was calculated by summing the cross-sectional areas of the ROIs across slices, and fat infiltration was determined based on the Hounsfield unit distribution within each ROI (Figure 1). Fat infiltration was calculated by analyzing the attenuation of muscle tissue on CT images. Skeletal muscle was defined using a Hounsfield unit (HU) range from −29 to +150, which has been widely applied in both cadaveric validation studies and clinical research [8,9,10,11]. This range differentiates skeletal muscle from adipose tissue (−190 to −30 HU) and has been used across diverse populations, including elderly cohorts and patients with spinal disorders, supporting its applicability to our study cohort. Nevertheless, we acknowledge that population-specific HU thresholds may provide additional accuracy, and future studies could explore tailored HU cut-offs for ADS patients. The proportion of muscle tissue within this HU range was then calculated as a percentage of the total muscle volume, providing the degree of FI [12].

**Figure 2:**
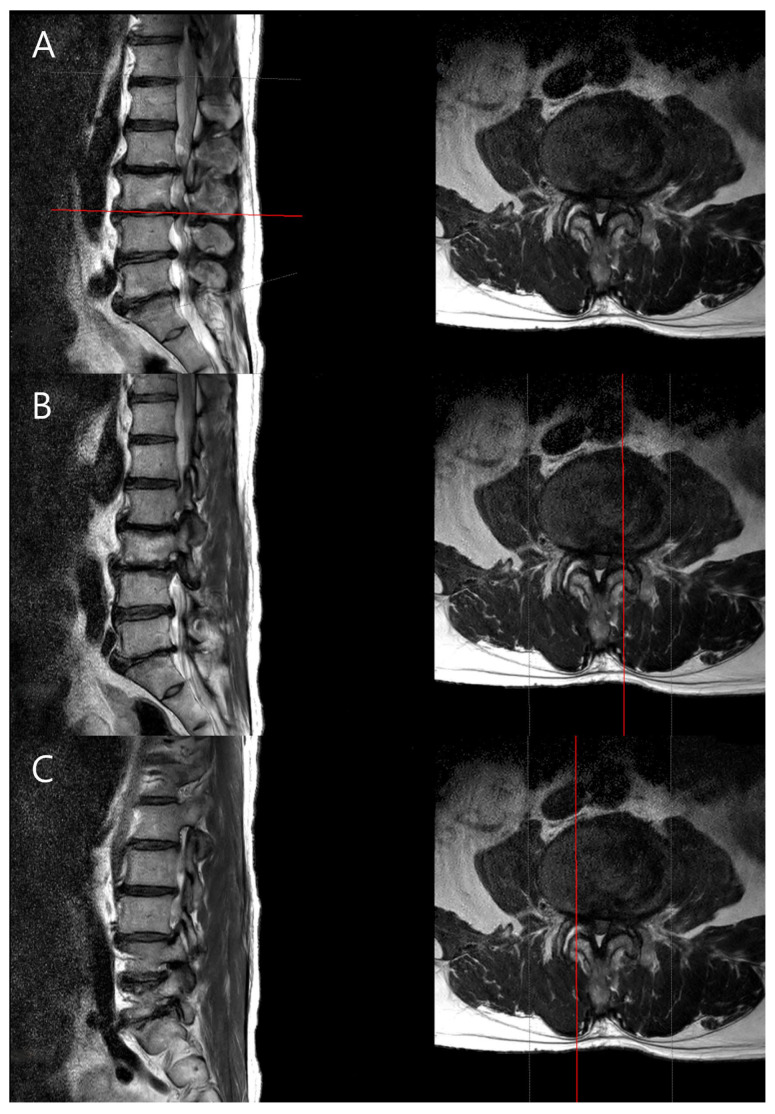
Evaluation of lumbar disc degeneration using T2 MRI images. (**A**) Axial plane showing the disc level. The horizontal red line on the sagittal image indicates the level of the axial cut shown on the right. (**B**,**C**) Sagittal and axial images were used to assess differences in disc degeneration between the concave and convex sides. The vertical red lines on the axial images delineate the sagittal cut plane, specifying whether it represents the concave or convex side for assessment.

Measurement of spinal parameters: The MRIs of the lumbar spine were performed on a 3.0-T scanner (SIGNA Premier, GE Healthcare, Chicago Illinois, USA) with a spine array coil. Intervertebral disc degeneration was graded using the Pfirrmann classification system based on T2-weighted sagittal images [13]. Foraminal stenosis was assessed using the Lee grading system, primarily on T1-weighted sagittal images with reference to T2-weighted sagittal images to exclude false-positive findings [14]. Facet joint degeneration was evaluated using the method described by Weishaupt et al., based on T2-weighted axial images [15]. Particularly, foraminal stenosis was assessed based on the radiological interpretations provided by the radiologist, ensuring consistency and accuracy in assessment. Typically, disc degeneration is graded based on the center of the disc, but to examine the differences between the CC and CV sides of the disc, we used the PACS viewer’s cross-link function to align the axial plane precisely with each disc level. From the disc’s midline, we moved laterally by three consecutive 2-mm axial slices (total 6 mm) to both the left and right sides, and the disc degeneration grade was determined in the sagittal plane for each cut. This standardized approach allowed us to assess the concave and convex sides separately (Figure 2).

Statistical analysis: All statistical analyses were performed using R software (version 4.0.5, R Foundation for Statistical Computing, Vienna, Austria). To assess normality, the Shapiro-Wilk test was applied to each variable. As the variables did not follow a normal distribution, the Wilcoxon signed-rank test was used to compare differences between the CC and CV sides for all measured variables. Differences between genders and curve directions were evaluated with the Mann-Whitney U test. Additionally, the association between the direction of scoliosis and the direction of symptoms was analyzed using the chi-square test. To ensure the reliability of muscle volume and fat infiltration measurements, two independent observers performed the measurements. Inter-observer reliability was assessed using the intraclass correlation coefficient (ICC) with a two-way random effects model based on absolute agreement. The analysis revealed a moderate level of reliability between the two observers, with an ICC of 0.678 (95% Confidence Interval, 0.530–0.784; *p* < 0.001). A *p*-value of less than 0.05 was considered statistically significant for all analyses. Multiple comparisons were corrected using a Bonferroni correction. Effect sizes were calculated to provide additional information on the magnitude of observed differences. According to recent guidelines [16], correlation coefficients (r) were interpreted as small (0.3), medium (0.5), and large (0.6).

## 3. Results

### 3.1. Patient Demographics and Overall Characteristics

**Figure 3:**
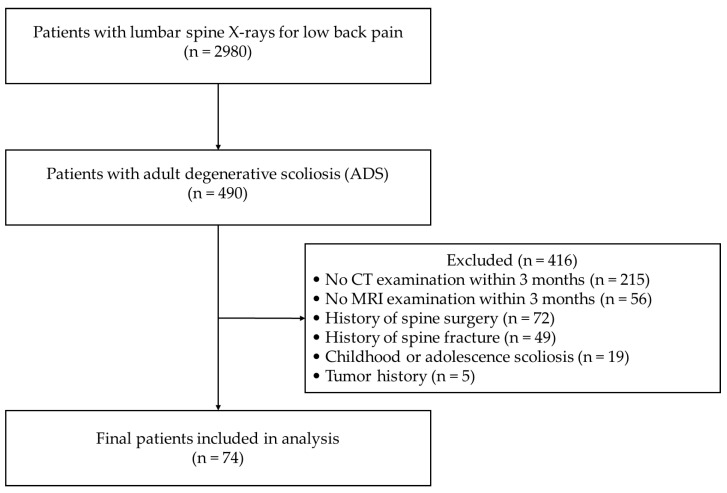
Consort flowchart of patient selection.

**Figure 4:**
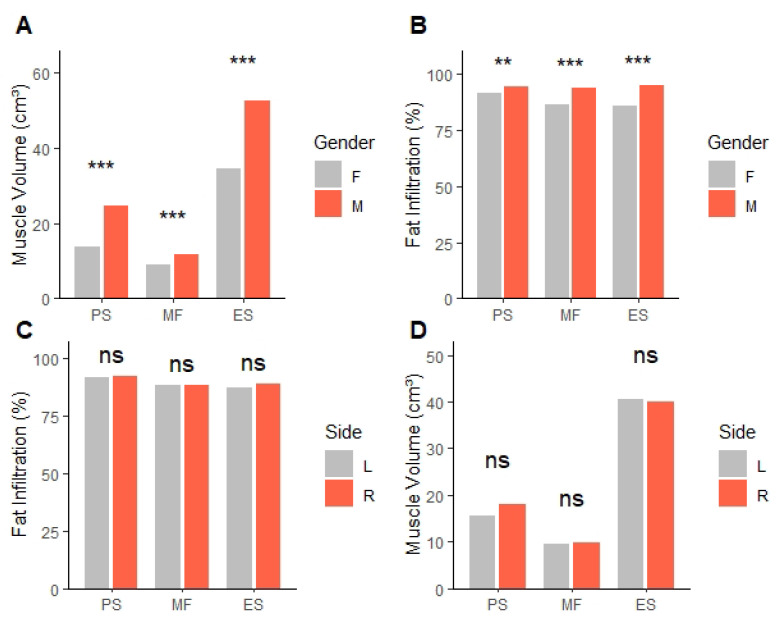
Subgroup analysis of muscle. (**A**,**B**) Muscle differences between females and males. (**C**,**D**) Muscle differences with the concave side direction. PS: psoas, MF: multifidus, ES: erector spinae, ns: not significant. ** *p* < 0.01 *** *p* < 0.001. Significant differences were evaluated by the Mann-Whitney U test.

A total of 2980 patients underwent lumbar spine X-rays for low back pain between January 2022 and December 2024. Among these, 490 were diagnosed with ADS. Following the predefined inclusion and exclusion criteria described in the Methods section, 74 patients were finally included in the analysis. The overall recruitment process is summarized in the CONSORT flow diagram (Figure 3). The patient group consisted of 51 females and 23 males, with a mean age of 71.4 years. The average Cobb angle of the patients was 14.44 degrees (Table 1). Most of the scoliosis curves had their apex at the L3 vertebra, observed in 51 patients, followed by L4 in 13 patients, L2 in 9 patients, and L5 in 1 patient. Regarding the convexity of the curves, 50 patients had their CC side on the right, while 24 patients had it on the left (Table 2). MV and FI were significantly different between males and females, but these differences were not influenced by whether the CC side was on the left or right (Table 3) (Figure 4). ES FI difference by gender showed borderline statistical significance (*p* = 0.019, r = 0.274), although it did not remain significant after Bonferroni correction (corrected α = 0.0167). No significant differences were observed in the PS FI and MF FI between males and females (Table 4).

### 3.2. MV and FI Differences Between CC and CV Sides

**Figure 5:**
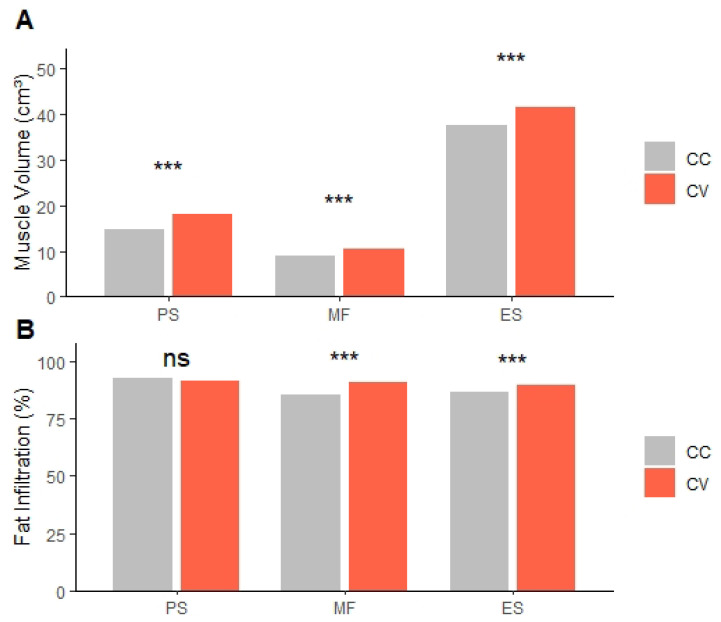
(**A**) MV difference between CC and CV Sides. (**B**) FI difference between CC and CV Sides. PS: psoas, MF: multifidus, ES: erector spinae, MV: muscle volume, FI: fat infiltration, CC: concave, CV: convex. ns: not significant. *** *p* < 0.001, Significant differences were evaluated by the Wilcoxon rank sum test.

The MV and FI of the PS, MF, and ES muscles are shown in Table 5 and Table 6. Comparing the CC and CV sides, the MV of PS, MF, and ES showed a significant difference between the two sides (*p* < 0.001). The FI of PS did not show a significant difference (*p* = 0.127). The FI of MF and ES showed a significant difference between the CC and CV sides (*p* < 0.001) (Figure 5).

### 3.3. Spinal Degenerative Changes Between CC and CV Sides

**Figure 6:**
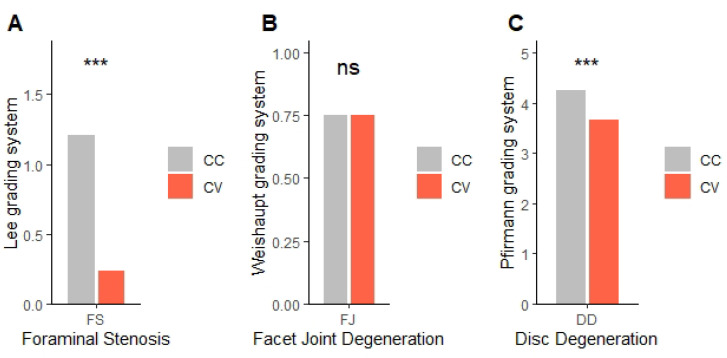
Spinal degenerative changes between CC and CV Sides. (**A**) Foraminal Stenosis, (**B**) Facet Joint Degeneration, (**C**) Disc Degeneration, CC: concave, CV: convex. ns: not significant. *** *p* < 0.001, Significant differences were evaluated by the Wilcoxon rank sum test.

The foraminal stenosis (FS), facet joint degeneration (FJD), and disc degeneration (DD) are shown in Table 7. Comparing the CC and CV sides, FS showed a significant difference between the two sides (*p* < 0.001). FJD did not show a significant difference (*p* = 0.842). DD showed a significant difference (*p* < 0.001) (Figure 6).

### 3.4. Relationship Between Curve Direction and Symptoms

Among the data analyzed from images of 74 patients, 5 patients were excluded because their exact symptoms were difficult to determine in their records, and only 69 patients were analyzed for the relationship between curve direction and symptoms. 89.9% of all patients (62) had radicular leg pain. Of these patients, 73.9% (51) had unilateral radicular leg pain. Twenty-seven patients had symptoms on the right side, twenty-four on the left side. Eleven patients had bilateral radiating pain. Seven patients simply complained of back pain and did not have radiating pain. A chi-square test showed no statistically significant relationship between the curve direction (left or right) and the side of the symptom (left, right, or both) (χ^2^ = 0.965, *p* = 0.326). This suggests that the curve direction is not associated with the side of symptoms in scoliosis patients. In other words, the results do not indicate whether symptoms are more likely to occur on the convex or concave side.

## 4. Discussion

In this study, we compared paraspinal MV, FI, and spinal degenerative changes between the CC and CV sides in patients with ADS. Our results showed that MV was consistently lower on the CC side across all examined muscles (PS, MF, ES). We also found that FI was significantly higher on the CC side for the MF and ES muscles, but not for the PS muscle. The difference in MV between CC and CV sides was observed in all PSM, including MF and ES, which are directly attached to the vertebral column, as well as in the psoas, which extends from the transverse process to the pelvis. Due to the difference in mechanical load between the CC and CV sides, this inevitably causes deformation in MV. However, differences in FI between sides were not observed in the PS but were present in the posterior PSM (MF and ES), suggesting that histological changes occurred only in the posterior PSM, not the PS. This assumption is supported by previous studies that identified histological changes, including degenerative changes and fat infiltration, especially in the posterior PSM. Also, there was a previous study that identified differential patterns of degeneration among PSM. A retrospective MRI-based study reported that FI in the posterior PSM was inversely correlated with FI in the PS [7,17,18,19,20]. This inverse correlation suggested a compensatory mechanism that weakening of the posterior PSM may result in strengthening of PS to maintain spinal stability. In addition, the large size of the PS could lead to less degeneration despite load imbalance. Future studies could test this hypothesis by investigating whether FI changes in PS occur asymmetrically as scoliosis progresses. Such studies may reveal the mechanisms of the progression of ADS, contributing to the development of targeted physical therapies for ADS.

In spinal degenerative changes, FS and DD were more severe on the CC side compared to the CV side, whereas FJD was not. The asymmetry in FS and DD might result from the uneven distribution of mechanical stresses in scoliotic spines. This finding is particularly important because it suggests that the CC side is at a higher risk for nerve root compression, as supported in previous literature [21,22]. The study by Liu et al. supports these observations [23]. They revealed that L3 and L4 nerve roots were predominantly compressed on the CC side of the scoliosis. However, as demonstrated in this study, clinical symptoms do not dominantly occur on the CC side. This suggests that nerve compression alone may not be the only cause of symptom development. Numerous previous studies using electromyography (EMG) in adolescent scoliosis patients have shown greater PSM activity on the CV side compared to the CC side, with the highest activity observed at the apex of the CV side [24,25]. Such excessive activation may increase muscle fatigue in the CV side, leading to clinical symptoms. Furthermore, a previous study reported that MV of PSM has no correlation with chronic low back pain, suggesting that multiple factors may contribute to symptom development in ADS [26]. In ADS, clinical symptoms are caused by a combination of nerve compression, muscle hypertonicity, and hyperactivity. This complexity makes it difficult to establish a consistent correlation between the direction of scoliosis and clinical symptoms.

In this study, we found the asymmetry in muscle and degenerative changes between the CC and CV sides. It remains unclear whether the asymmetry of these spinal structures initiated the development of ADS or whether it emerged as a consequence of ADS progression. Although the causality remains uncertain, the presence of structural asymmetry suggests that targeted physiotherapy focused on CC side muscle strengthening may help restore muscle balance and reduce the progression of degenerative changes. Certain rehabilitation protocols, including neuromuscular stimulation or resistance training aimed at the CC side, could potentially improve muscle quality and function, slowing the progression of scoliosis. A study by Ryu et al., using sEMG data and radiological analysis, proposed an exercise protocol specifically targeting the CC side muscle strengthening in patients with scoliosis [27]. The conclusion was that strengthening the PSM on the CC side could improve the severity of scoliosis.

There are some limitations in this study. This study is a cross-sectional design that cannot establish causal relationships or changes over time. Longitudinal studies are needed to understand the progression of degeneration in ADS patients. We analyzed a relatively small sample size, which allows for the detection of only large effects with 90% power according to recent guidelines [16]. Therefore, future studies with larger sample sizes are needed to validate and extend our findings. In addition, because MV and FI were measured manually, it takes much time, and there is potential for measurement errors. This limitation could be addressed in future studies by employing artificial intelligence (AI) based image analysis techniques. This study focused on patients who visited the hospital with symptomatic pain, excluding asymptomatic scoliosis patients. Thus, the findings may not be generalized to the broader scoliosis population and suggest a potential selection bias. With electromyography, we can assess muscle function directly. By combining structural and functional assessments, we can provide a more comprehensive understanding of muscle changes in ADS.

## 5. Conclusions

In patients with Adult degenerative scoliosis, we found that muscle volume was consistently lower on the concave side across all paraspinal muscles, whereas fat infiltration in the multifidus and erector spinae was higher on the concave side. Degenerative changes in the spine, such as foraminal stenosis and disc degeneration, were also more severe on the concave side, although facet joint degeneration did not. These results suggest that spinal muscle and structural degeneration are not evenly distributed between the two sides. Physiotherapy that specifically targets the more affected concave side may offer better outcomes for patients with adult degenerative scoliosis. Future studies should focus on the longitudinal progression of this asymmetry and its relationship with clinical outcomes. Additionally, advanced imaging technologies may further enhance our understanding of Adult degenerative scoliosis pathology and its clinical implications.

## Figures and Tables

**Table 1:** Patient demographics.

Number of Cases	74
Gender (male/female)	23/51
Age (year)	71.38 ± 8.6
Cobb angle (°)	14.44 ± 4.6

**Table 2:** Subgroup analysis of Cobb angle.

	Number	Cobb Angle
Gender		
Male	23	11.71 [11.43, 12.44]
Female	51	13.35 [12.34, 15.10]
*p*		0.029
Effect size (r)		0.319
CC		
Right	50	12.57 [11.75, 14.61]
Left	24	12.44 [11.11, 14.10]
*p*		0.617
Effect size (r)		0.072

**Table 3:** FI difference between male and female groups by muscle.

	Male	Female	*p*	Effect Size (r)
PS FI	95.02 [94.10–96.95]	92.35 [91.00–94.25]	0.0095	0.386
MF FI	95.12 [92.78–96.95]	88.07 [85.60–90.43]	<0.001	0.635
ES FI	95.18 [93.95–97.05]	91.28 [85.80–92.90]	<0.001	0.647

**Table 4:** FI difference between CV and CC side by gender.

Difference	Male	Female	*p*	Effect Size (r)
PS FI	−2.35	−1.6	0.089	0.200
MF FI	2.6	5.92	0.068	0.215
ES FI	1.2	4.06	0.019	0.274

**Table 5:** MV difference between CC and CV sides (cm^3^).

	Concave	Convex	*p*	Effect Size (r)
PS MV	14.20 [11.60, 15.90]	16.85 [15.00, 19.25]	<0.001	0.934
MF MV	8.25 [7.15, 9.65]	10.50 [9.40, 12.00]	<0.001	0.846
ES MV	34.05 [31.35, 39.50]	38.35 [35.80, 44.60]	<0.001	0.728

**Table 6:** FI difference between CC and CV sides (%).

	Concave	Convex	*p*	Effect Size (r)
PS FI	93.35 [92.40, 95.05]	93.25 [91.65, 94.70]	0.24	0.211
MF FI	86.85 [84.90, 91.00]	93.80 [90.40, 95.20]	<0.001	0.703
ES FI	91.15 [88.30, 92.50]	93.95 [91.40, 95.20]	<0.001	0.583

**Table 7:** Spinal degenerative changes between CC and CV sides.

	Concave	Convex	*p*	Effect Size (r)
FS	1.21 [1.00, 1.42]	0.24 [0.11, 0.36]	<0.001	0.896
FJD	0.75 [0.61, 0.89]	0.75 [0.61, 0.89]	0.842	0.000
DD	4.26 [4.10, 4.43]	3.67 [3.55, 3.78]	<0.001	0.782

## Data Availability

The data presented in this study are available on request from the corresponding author.

## Abbreviations

The following abbreviations are used in this manuscript:

ADS	Adult degenerative scoliosis
PSM	paraspinal muscle
MF	Multifidus
ES	erector spinae
PS	Psoas
MV	muscle volume
FI	fat infiltration
CC	Concave
CV	Convex
FJD	facet joint degeneration
DD	disc degeneration
FS	foraminal stenosis
